# FSCN1-mediated hepatic gluconeogenesis is indispensable for neonatal mice survival: FSCN1 regulates gluconeogenesis and neonatal survival

**DOI:** 10.3724/abbs.2025146

**Published:** 2025-08-29

**Authors:** Xiangxiang Liu, Yuanzhao Hu, Liangwei Wu, Yiwen Zhang, Lei Sang, Yake Gao, Lei He, Wenyong Xiong, Shengyu Yang, Jianwei Sun

**Affiliations:** 1 Yunnan Key Laboratory of Cell Metabolism and Diseases Center for Life Sciences School of Life Sciences Yunnan University Kunming 650500 China; 2 Key Laboratory of Medicinal Chemistry for Natural Resource Ministry of Education; Yunnan Key Laboratory of Research and Development for Natural Products; School of Pharmacy Yunnan University Kunming 650500 China; 3 Department of Cell and Biological System Penn State Cancer Institute Penn State College of Medicine Hershey PA 17033 USA

**Keywords:** FSCN1, neonatal mice survival, hypoglycemia, gluconeogenesis

## Abstract

Actin-bundling protein Fascin1 (FSCN1) is encoded by the *Fscn1* gene and is crucial for cytoskeletal remodeling and cellular migration. Although a previous study linked *Fscn1* deficiency to neonatal lethality in mice, the underlying metabolic mechanism remains unclear. In this study, we report that systemic knockout (KO) of *Fscn1* leads to 52.2% mortality within 24 h post-birth, accompanied by severe hypoglycemia in KO pups compared with their littermates. Remarkably, this lethality is fully rescued by oral glucose administration, indicating a glucose supply-dependent survival mechanism. Surviving *Fscn1*-KO neonates display persistent developmental deficits, including growth retardation and depleted lipid stores, despite intact canonical insulin-regulated hepatic gluconeogenic pathways. Transcriptomic profiling of P0 livers reveals that *Fscn1* loss predominantly disrupts metabolic pathways, with the glycerol phosphate shuttle being the most significantly downregulated module. Mechanistically, *Fscn1*-KO livers exhibit markedly reduced protein levels of glycerol-3-phosphate dehydrogenase isoforms (GPD1/GPD2), key enzymes bridging glycolysis and gluconeogenesis. Consistently, glycerol tolerance tests demonstrate impaired glycerol-to-glucose conversion in *Fscn1*-KO mice, confirming defective glycerol-driven gluconeogenesis. Our findings establish FSCN1 as a novel cytoskeletal-metabolic integrator essential for neonatal survival by sustaining hepatic glucose production from glycerol, thus revealing an unexpected role of actin dynamics in coordinating metabolic adaptation during early postnatal development.

## Introduction

*Fscn1* is a pro-metastasis gene that encodes the actin-bundling protein Fascin1 (also named as Fascin). Fascin1 crosslinks filamentous actin (F-actin) to promote the formation of invasive protrusions that facilitate cell migration and invasion [ 1– 4] . In addition to its classical roles, FSCN1 modulates actin-binding proteins [ 5– 7] , interacts with microtubules [8], and integrates with the linker of the nucleoskeleton and cytoskeleton complex [9]. While the oncogenic roles of FSCN1 in cancer progression and metabolic homeostasis are well documented [ 10– 19] , its physiological functions remain poorly understood.

In adult tissues, FSCN1 is expressed at low levels in dendritic cells, neurons and vascular endothelial cells, whereas its expression peaks during embryonic development, particularly in the neural and mesenchymal lineages [1]. Although murine studies initially suggested that FSCN1 is dispensable during development [ 20, 21] , *Fscn1* deficiency causes neonatal lethality post-birth without affecting embryo viability [22]. It was hypothesized that this perinatal lethality was due to postnatal feeding or respiratory challenges in *Fscn1*-KO pups [22]. More recently, *Fscn1*-KO females were reported to have lactation defects and feeding abnormalities [23].

Hepatic gluconeogenesis involves the synthesis of glucose from non-carbohydrate precursors such as glycerol, lactate, and amino acids [ 24, 25] . While adults rely on oxaloacetate-mediated gluconeogenesis through phosphoenolpyruvate carboxykinase 1 (PCK1) [ 26, 27] , neonates prioritize a glycerol-centric gluconeogenesis pathway to take advantage of lipid-rich maternal milk. This pathway begins with glycerol phosphorylation by glycerol kinase (GK), followed by conversion to dihydroxyacetone phosphate (DHAP) via glycerol-3-phosphate dehydrogenase (GPD1/GPD2), ultimately fueling gluconeogenesis [28]. Neonates undergo rapid metabolic shifts post-birth, marked by elevated glucagon and decreased insulin levels, which rapidly activate hepatic gluconeogenesis to meet the high glucose demand of the brain [29]. Importantly, neonatal gluconeogenesis contributes 8%–9% of total glucose production—a threshold indispensable for survival [30]. Despite evidence linking *Fscn1* deficiency to neonatal lethality via hypoglycemia, the mechanistic basis of this metabolic dysregulation remains unresolved. This study investigated whether impaired gluconeogenic capacity underlies the hypoglycemia-driven mortality observed in *Fscn1*-deficient neonates.

Herein we demonstrate that FSCN1 is indispensable for neonatal hepatic gluconeogenesis. Postnatal *Fscn1*-KO mice presented severe metabolic dysregulation characterized by persistent hypoglycemia and impaired glycerol-driven gluconeogenesis. Lethality in *Fscn1*-KO mice can be rescued through glucose supplementation. These findings indicate that FSCN1 orchestrates metabolic reprogramming during the neonatal adaptation response, establishing its essential role in maintaining hepatic glucose production and ensuring newborn survival.

## Materials and Methods

### Animals

All animal experiments were conducted in accordance with protocols approved by the Institutional Animal Care and Use Committee of Yunnan University (Ethical Committee Approval No. SINH-2020-DQR-3). Wild-type C57BL/6J mice were obtained from the Animal Center of Yunnan University. An *Fscn1*-knockout mouse model was generated with a deletion of 543 bp across 2 exons and maintained under a specific pathogen-free (SPF) condition.

Embryonic specimens were collected from timed-pregnant *Fscn1* ^*+*/ *–*^ dams at embryonic day 19 (E19) via uterine dissection. A heterozygous intercross breeding strategy ( *Fscn1* ^*+*/ *–*^  ♀ × *Fscn1* ^*+*/ *–*^♂) was implemented to analyze Mendelian inheritance patterns, with neonatal genotypes determined via PCR amplification [31] using primers listed in Supplementary Table S1, and quantified for distribution analysis. Postnatal day 0 (P0) littermates were utilized for dual-omics profiling (transcriptomics and metabolomics), whereas 6-week-old littermates were assessed for growth retardation phenotypes through longitudinal body weight measurements. Metabolic evaluations, including insulin tolerance tests (ITT) and glycerol gluconeogenesis assays (glycerol tolerance tests), were performed on P35 mice (see Supplementary Table S2 for reagents). Age-matched (± 3 days) littermate pairs were prioritized for comparative analyses to control for environmental variability. Sex determination via *Sry* gene analysis [32] confirmed that there were no sex-based disparities in glucose levels or ATP production ( *P*  > 0.05). Wild-type (WT) controls were generated from an independent C57BL/6J breeding colony to ensure that there were enough experimental animals, as the number of WT littermates from the heterozygous crosses was lower.

### Cell culture and genetic manipulation

Mouse embryonic fibroblasts (MEFs), human embryonic kidney 293T (293T) cells, and a human hepatocellular carcinoma cell line (Huh7) were acquired from the American Type Culture Collection (ATCC, Manassas, USA). All the cell lines were maintained as adherent cultures in DMEM (Gibco, Shanghai, China) supplemented with 10% fetal bovine serum (ExCellBio, Suzhou, China) and 1% penicillin/streptomycin (Solarbio, Beijing, China) under standard conditions (37°C, 5% CO _2_) without mycoplasma contamination. For genetic perturbation, shRNA constructs targeting *Fscn1* (sh *Fscn1*) were subcloned and inserted into the pLKO.1-TRC vector, while CRISPR/Cas9 guide RNAs (sg *Fscn1*) were engineered into the Lenti-CRISPR-V2 backbone. The oligonucleotide sequences used for RNA interference and gene editing are shown in Supplementary Table S3, with vector construction protocols adapted from established methodologies ( https://www.addgene.org/protocols/plko/ and https://www.zlab.bio/guide-design-resources). All expression vectors were subjected to Sanger sequencing verification (Tsingke, Beijing, China) prior to lentiviral packaging and cellular transduction.

### Glucose measurement and metabolic challenge tests

Neonatal blood samples (P0) were collected in 2% EDTA-coated tubes (Biofil, Guangzhou, China) and centrifuged at 684 *g* for 10 min at 4°C to isolate the plasma. Quantitative glucose analysis was performed via a glucose oxidase-based assay kit (Nanjing Jiancheng Bio, Nanjing, China), and the detailed methods were as follows: 5 μL plasma aliquots were combined with 200 μL reaction reagent in 96-well plates (Biofil), incubated at 37°C for 10 min, and the absorbance was measured at 505 nm via a microplate reader (Biotek, Winooski, USA) with ddH _2_O serving as a negative control. For fasting studies, age-matched WT, *Fscn1* ^*+*/ *–*^ and *Fscn1*-KO mice underwent controlled fasting (16 h: 18:00–10:00) with ad libitum access to water, followed by paired fed/fasted blood sampling via tail vein puncture. Glycerol tolerance tests (glycerol-mediated gluconeogenesis tests) were conducted on P35 mice from the *Fscn1* ^*+*/ *–*^ and *Fscn1* ^*–*/ *–*^ groups following 16 h of overnight fasting. After baseline glucose measurement, the mice were numbered, weighed, and orally gavaged with 2.5% glycerol solution (2.73 mM in saline; Sangon Biotech, Shanghai, China) at 10 mL/kg body weight on the basis of methods referenced for glycerol quantification [ 27, 33] . Serial glucose measurements were monitored at 0, 30, 60, 90, and 120 min post-administration using standardized tail nick procedures. The area under the curve (AUC) was calculated via the trapezoidal method. All experimental protocols were synchronized with facility light cycles (06:00–18:00) to minimize circadian variability.

### Multi-omics profiling

Neonatal littermates ( *Fscn1* ^*+*/ *–*^ vs *Fscn1* ^*–*/ *–*^) were genotyped within 24 h postpartum via tail biopsy PCR prior to hepatic tissue collection. Liver samples ( *n*  = 3‒4 per group) were homogenized in TriZol reagent (Lablead, Beijing, China) and stored at –80°C until RNA extraction. Paired-end RNA sequencing libraries were prepared and constructed at Novogene (Beijing, China) with a read length of 2 × 150 bp on a NovaSeq 6000 platform (Illumina, San Diego, USA). Both the raw data and the clean data were subjected to FastQC v0.11.9 quality control, and index construction was performed with Bowtie2 ( http://bowtie-bio.sourceforge.net/Bowtie2/index.shtml), while genome alignment of the clean data was conducted via TopHat2 ( https://ccb.jhu.edu/software/tophat/index.shtml). Transcriptome assembly was carried out via Cufflinks ( https://cole-trapnell-lab.github.io/cufflinks/). Gene expression levels were calculated via the fragments per kilobase million reads (FPKM) method. A *t* test was used to calculate *P* values for differentially expressed genes (DEGs) with | log2 (fold change) | ≥ 1, considering *P* values < 0.05 as significant. The raw transcriptome data were obtained, and subsequent analysis was performed as previously described [34].

Untargeted metabolomics analysis employed liquid nitrogen-snap-frozen P0 hepatic samples ( *n* = 3 per group) processed through cryogenic milling, methanol:acetonitrile:water (2:2:1) extraction and lyophilization, which strictly followed the Agilent metabolomics sample preparation procedure [35]. The reconstituted extracts were analyzed on an Agilent 6545 Q-TOF LC/MS system equipped with an Agilent SB C18 column (2.1 × 100 mm, 1.8 μm; Agilent, Santa Clara, USA) via gradient elution, with data acquired in positive/negative ESI mode and solvent blanks used as procedural controls.

### Immunoblotting

Liver tissues were dissected and immediately homogenized in ice-cold RIPA lysis buffer (Beyotime Biotechnology, Nanjing, China) containing a protease/phosphatase inhibitor cocktail (MCE, Shanghai, China). After a 1-h incubation on ice, the lysates were centrifuged at 11,000 *g* for 15 min at 4°C. Protein quantification was performed via a BCA kit (Thermo Fisher Scientific, Shanghai, China) with bovine serum albumin standards. After protein denaturation with loading buffer, 10%–12.5% gradient SDS-polyacrylamide gels (Epizyme Biotech, Shanghai, China) were used for immunoblotting experiments. Following wet transfer, the PVDF membranes (Millipore, Shanghai, China) were blocked with 5% non-fat milk in TBS-T (Tris-buffered saline with 0.1% Tween-20) for 1 h at room temperature, followed by overnight incubation at 4°C with the following primary antibodies: anti-GPD1 (ABclonal, Wuhan, China) and anti-GPD2 (Proteintech, Wuhan, China). After three times of wash with TBS-T, the membranes were incubated with HRP-conjugated secondary antibodies for 1 h at 4°C. The protein blots were visualized via an enhanced chemiluminescence (ECL)-plus Western blotting detection system (Tanon-5200 Multi; Tanon, Shanghai, China). The primary antibody was diluted to 1:1000, and the secondary antibody was diluted to 1:10,000. Additionally, detailed information regarding the antibodies used is provided in Supplementary Table S4.

### RNA isolation and real-time quantitative PCR

Total RNA was extracted from hepatic tissues and cell lines via TriZol reagent (Lablead, Beijing, China). The RNA purity and concentration were determined spectrophotometrically (A260/A280 ratio of 1.8–2.0) with a NanoDrop 2000 (Thermo Fisher Scientific). Reverse transcription was performed with 1 μg of RNA via the First-strand cDNA Synthesis Mix (Lablead). Quantitative PCR amplification was conducted in triplicate via a 2× Realab Green PCR Fast mixture (Lablead) on a Real-Time PCR System (Bio-Rad, Hercules, USA) under the following cycling conditions: initial denaturation at 95°C for 2 min; 40 cycles of 95°C for 10 s and 60°C for 30 s; and melt curve analysis (65–95°C, 0.5°C/s increments). Gene expression quantification was performed via the 2 ^–ΔΔCt^ method [36]. The sequences of the primers used are listed in Supplementary Table S1.

### Pathological examination and immunity-related experiments

Hepatic samples were immediately fixed in 4% paraformaldehyde (PFA) in phosphate-buffered saline (pH 7.4) for 24 h at 4°C. The fixed tissues were dehydrated with graded ethanol (70%–100%), cleared with xylene, and embedded in paraffin. Serial 5-μm sections were cut with a rotary microtome (Leica, Wetzlar, Germany) and mounted on poly-L-lysine-coated slides (Citotest, Nanjing, China). Hematoxylin and eosin (H&E) staining was performed according to previously established procedures [37]. Specifically, H&E staining solution was applied for 2 min, followed by differentiation via an acidic differentiation solution for 5 s, after which the sections were washed for 10 min to achieve blue coloration. After a series of ethanol dehydration and xylene clearing steps, 2–3 drops of neutral gum were immediately added to the sections, which were then sealed and dried at 37°C. Finally, the slides were scanned for pathological analysis by whole slide scanning system (Teksqray, Shenzhen, China).

### Measurement of lipid-related components

Serum and hepatic lipid profiles were comprehensively analyzed in WT, *Fscn1* ^*+*/ *–*^, and *Fscn1* ^*–*/ *–*^ mice at P0 and P35. Serum samples were centrifuged at 684 *g* for 15 min at 4°C, while liver homogenates were prepared through sequential centrifugation at 11,000 *g* for 15 min twice at 4°C. The levels of non-esterified fatty acids (NEFA) in the serum and liver tissues were quantified via an automatic biochemical analyzer following the manufacturer’s instructions. Glycerol levels were assessed via a glycerol assay, with lipid components, including triglyceride (TG), total cholesterol (T-CHO), high-density lipoprotein cholesterol (HDL-C), and very low-density lipoprotein cholesterol (VLDL-c), measured under standardized conditions. The hepatic ketone bodies β-hydroxybutyrate (BOH) and acetoacetate (AcAc) were analyzed via a ketone assay kit. All metabolite concentrations were normalized to the total protein content determined via a BCA assay kit, and the experimental details of the commercial kits are provided in Supplementary Table S2.

### Insulin level and insulin tolerance test

To investigate the effects of insulin signaling on blood glucose levels, insulin levels were determined and insulin tolerance test (ITT) were conducted. Owing to the limited serum volume from P0 neonates, systemic insulin levels were evaluated in P35 mice ( *Fscn1* ^*+*/ *–*^ vs *Fscn1* ^*–*/ *–*^) following a 4-h fasting (08:00–12:00). Blood was collected via retro-orbital puncture into serum separator tubes. Insulin concentrations were determined via a mouse-specific ELISA kit (Jonln Biotechnology, Shanghai, China). For the insulin tolerance test, age-matched mice (5 weeks old) were fasted for 4 h (08:00–12:00) with free access to water, and then weighed. After baseline glucose measurement, the mice were intraperitoneally injected with recombinant human insulin (Novo Nordisk, Copenhagen, Denmark) at 0.5 U/kg body weight. Serial glucose measurements were performed via tail vein sampling at 0, 15, 30, 60, and 120 min post-injection.

### Statistical analysis

The experimental cohorts were randomized from standardized breeding litters. No statistical methods were employed to predetermine sample sizes. Compared with *Fscn1* ^*+*/ *–*^ mice, 462 genes with a |log2FoldChange| ≥ 1 and a *P* value < 0.05 were screened by using the R software limma package for differential analysis [38]. Quantitative densitometry of the western blot bands was performed using Image J software. All datasets were analyzed using GraphPad Prism 9.0.1 and are presented as the mean ± SEM (standard error of the mean) from *n* ≥ 3 independent biological replicates. Intergroup comparisons were performed using two-tailed unpaired Student’s *t* tests, with *P* values less than 0.05 considered significant. Survival curves were generated via Kaplan-Meier methodology.

## Results

### *Fscn1* deficiency triggers hypoglycemia-induced neonatal lethality

**Figure 1:**
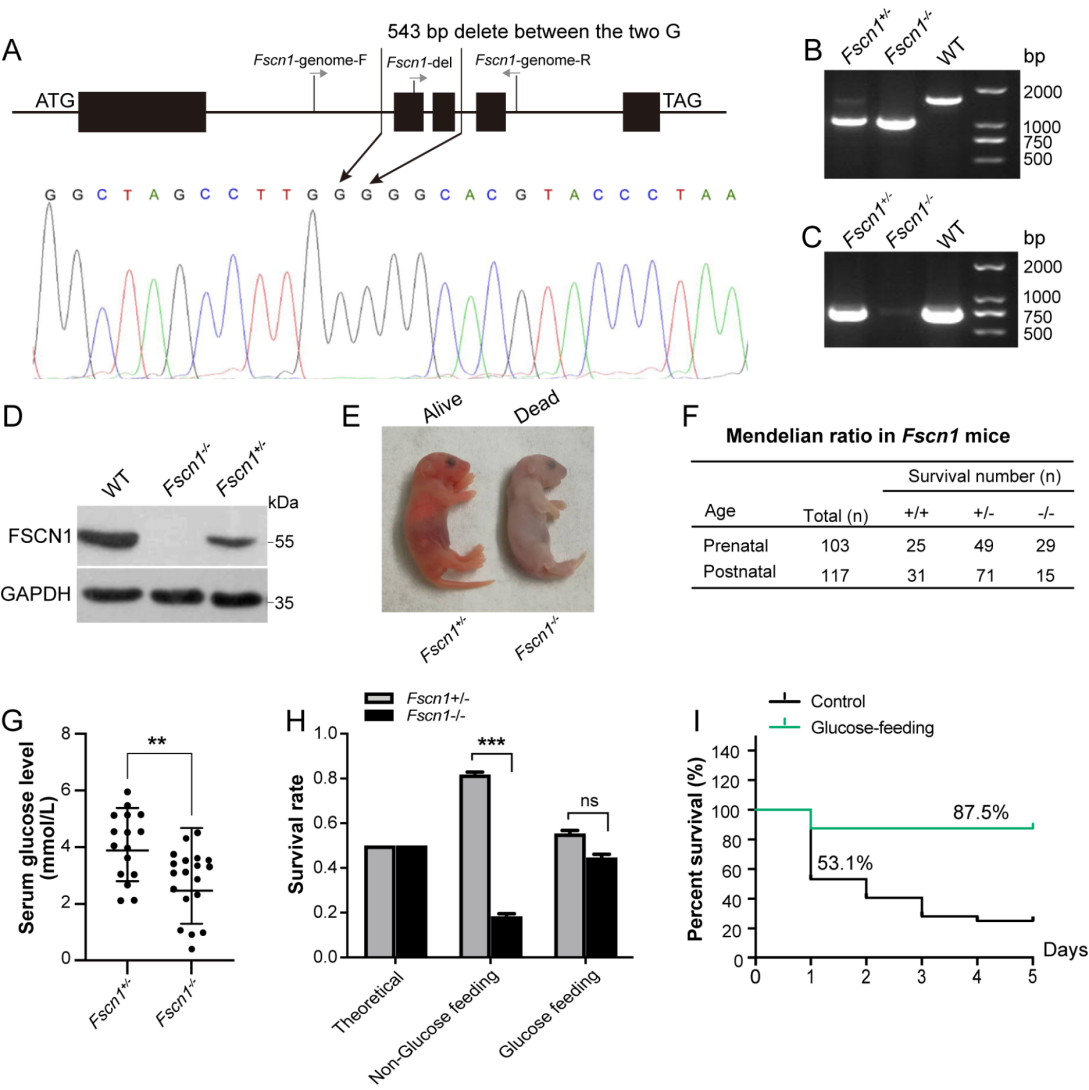
Figure 1 Systematic knockout of *Fscn1* induces hypoglycemia and high neonatal mortality (A) Schematic diagram illustrating the construction of a systemic Fscn1 knockout. (B) PCR analysis confirming WT mice, indicated by a 1620 bp band, via the primers Fscn1-genome-F and Fscn1-genome-R. (C) PCR analysis identifying Fscn1 –/– mice, characterized by the absence of a 739 bp band, via the primers Fscn1-del and Fscn1-genome-R. (D) Western blotting verification of the absence of FSCN1 protein in the liver tissue of knockout mice. (E) Representative image showing Fscn1-KO neonates that died within 24 h after birth. (F) Statistical analysis of Fscn1 +/– offspring ratios obtained via heterozygous mating (♀Fscn1 +/– & ♂ Fscn1 +/– ). (G) Serum blood glucose levels in Fscn1 +/– and Fscn1 –/– P0 mice (n = 16 for Fscn1 +/– ;n = 19 for Fscn1 –/– ). (H) Offspring survival ratios of Fscn1 +/– and Fscn1 –/– P0 mice after oral glucose supplementation within 24 h post-birth (glucose content of 100 g/L). Lethality rate of nonglucose-fed Fscn1 –/– mice: 47.8% (ratio, 11/23); lethality rate of glucose-fed Fscn1 –/– mice: 20% (ratio, 4/20). (I) Survival curves of Fscn1 +/– and Fscn1 –/– mice (P0) within the first 5 days postnatally, with or without oral glucose supplementation (n = 32 for the control; n = 16 for the glucose-fed group). The mice used in these experiments were littermates. Data are presented as the mean ± SEM, and statistical significance was determined via two-tailed t tests; *P < 0.05, **P < 0.01, ***P < 0.001, and ns means P > 0.05.

To investigate the physiological role of FSCN1, we generated a systemic *Fscn1*-knockout (KO) mouse model ( Figure 1A). Genotyping confirmed a 543 bp deletion spanning two *Fscn1* exons. PCR amplification via the primer pairs *Fscn1-genome-*F and *Fscn1-genome-*R produced a single band in WT mice, whereas the heterozygous *Fscn1* ^*+*/ *–*^ gene exhibited dual amplicons (1620 bp and 1077 bp) corresponding to WT and *Fscn1-*KO mice ( Figure 1B). *Fscn1*-KO mice were identified via the deletion-specific primer pair *Fscn1-del* and *Fscn1-genome-*R, which produces a single band (739 bp) in WT and heterozygous mice but no band in homozygous *Fscn1*-KO mice ( Figure 1C). Western blot analysis further confirmed the absence of FSCN1 in *Fscn1*-KO mice ( Figure 1D).

Strikingly, *Fscn1* ^*–*/ *–*^ neonates exhibited postnatal lethality within 24 h after birth ( Figure 1E). To further investigate these findings, we employed a mating strategy involving female/male heterozygotes (♀ *Fscn1* ^*+*/ *–*^ & ♂ *Fscn1* ^*+*/ *–*^). Upon examination of the plug in the fertilized female, we focused on mice in the embryonic period of E18.0 (Prenatal) and within 24 h of birth (Postnatal). Genotypic analysis of prenatal mice revealed that heterozygous offspring nearly adhered to the Mendelian inheritance ratio of 1:2:1 (WT: *Fscn1* ^*+*/ *–*^: *Fscn1* ^*–*/ *–*^ = 25:49:29) ( Figure 1F). Notably, 47.8% of *Fscn1* ^*–*/ *–*^ neonates died postnatally during the first 24 h of life ( Supplementary Figure S1A), indicating that *Fscn1* deficiency-induced neonatal death predominantly occurred in the postnatal period within 24 h, which is consistent with previous reports [22].

Given the importance of the energy supply for neonatal survival, we measured serum and liver glucose levels. *Fscn1*-KO neonates presented severe hypoglycemia, with significantly reduced glucose concentrations in both serum (2.85±0.28 mM *vs*. 4.07±0.30 mM in *Fscn1* ^*+*/ *–*^ littermates) and liver homogenate ( Figure 1G and Supplementary Figure S1B). Consistent with the role of AMPK in glucose sensing [39], *Fscn1*-KO neonates presented increased hepatic AMPK phosphorylation at Thr192, a hallmark of energy stress, accompanied by decreased ATP levels ( Supplementary Figure S1C,D), indicating energy deprivation due to *Fscn1* deficiency. Notably, oral glucose supplementation (100 g/L) increased survival rates from 53.1% to 87.5% ( Figure 1H,I), directly linking neonatal lethality to glucose insufficiency. The mortality of *Fscn1* ^*–*/ *–*^ P0 mice decreased from 47.8% to 20% after glucose supplementation ( Supplementary Figure S1A). These findings identify hypoglycemia as the primary driver of neonatal lethality in *Fscn1*-deficient mice, establishing glucose insufficiency as the central metabolic defect in this model.

### Postnatal hypoglycemia drives developmental defects independent of insulin signaling

**Figure 2:**
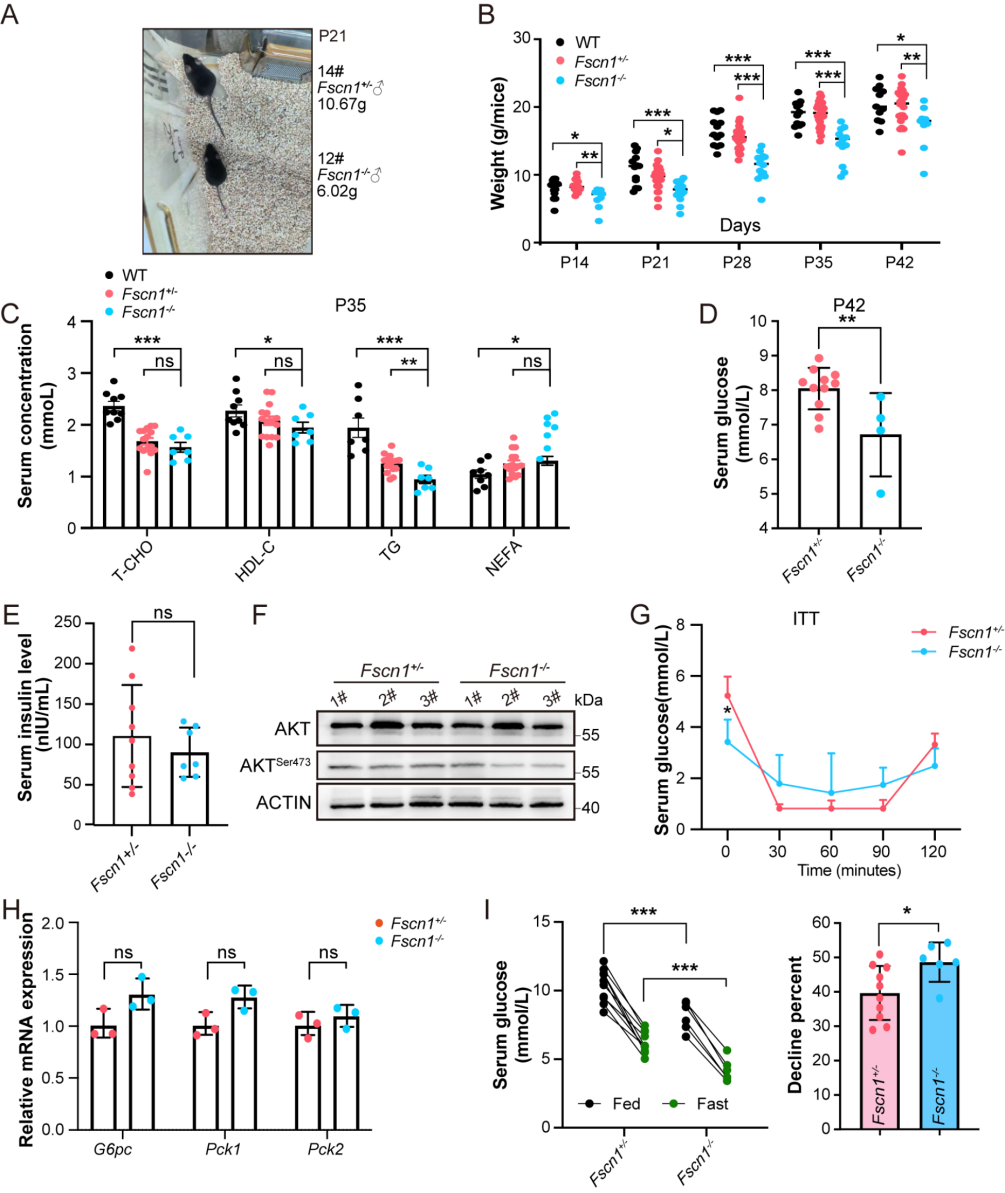
Figure 2 *Fscn1* deficiency induces persistent hypoglycemia and growth retardation in mice (A) Images depicting body size differences between Fscn1 +/– and Fscn1 –/– mice at postnatal day 21 (P21), highlighting differences in overall growth. (B) Body weight measurements of Fscn1 +/– and Fscn1–/– mice from 2–6 weeks (each group, n ≥ 9). (C) Lipid profile analysis of P35 mice, including T-CHO, HDL-C, tTG and NEFA (each group, n ≥ 7, except NEFA measurements, which were performed on 3 mice per group). (D) Serum glucose levels in Fscn1 +/– and Fscn1 –/– P42 mice (Fscn1 +/– ,n = 11; Fscn1 –/– ,n = 4). (E) Serum insulin concentrations in P35 mice (Fscn1 +/– ,n = 9; Fscn1 –/– ,n = 7). (F). Western blot analysis of AKT phosphorylation in Fscn1 +/– and Fscn1 –/– P0 livers (each group, n = 3). (G) Insulin tolerance test in P35 mice administered 0.5 U/kg insulin after 4 h of fasting (each group, n ≥ 6). (H) Relative mRNA levels of key gluconeogenesis-related genes (G6pc,Pck1, and Pck2) in the livers of Fscn1 +/– and Fscn1 –/– mice at P0 (each group, n = 3). (I) Effects of starvation on blood glucose utilization in P35 mice (each group, n ≥ 6). All the mice used in these experiments were littermates, except the WT mice. Data are presented as the mean ± SEM, and statistical significance was determined via two-tailed t tests; *P < 0.05, **P < 0.01, ***P < 0.001, and ns means P > 0.05.

To evaluate the long-term effects of *Fscn1* deficiency, we longitudinally monitored the body weights of untreated littermates from postnatal day 14 (P14) to P42. To minimize neonatal stress, the mice were identified via toe marking at P14. Compared with *Fscn1* ^*+*/ *–*^ littermates, *Fscn1*-KO mice presented pronounced growth impairment, with significantly reduced body size and persistent weight deficits (P14--P42) ( Figure 2A,B). Given the association between dysregulated cholesterol metabolism and growth retardation [ 40, 41] , we examined lipid levels in P35 mice. Surprisingly, *Fscn1*-KO mice displayed systemic hypolipidemia, characterized by reductions in T-CHO, HDL-C, NEFA and TG levels, relative to those of their WT and *Fscn1* ^*+*/ *–*^ counterparts ( Figure 2C). This broad range of lipid depletion suggests that global metabolic dysfunction underlies the growth phenotype. Notably, *Fscn1*-KO mice maintained chronically depressed blood glucose levels into adulthood (6 weeks old, Figure 2D), indicating a developmental failure to establish glucose homeostasis. The persistence of hypoglycemia beyond the neonatal period underscores the essential role of FSCN1 in metabolic programming during early development and warrants further investigation of insulin signaling dynamics.

Hyperinsulinemic hypoglycemia (HH), typically characterized by high insulin secretion during hypoglycemia [42], was excluded as a mechanistic contributor in *Fscn1*-KO mice. The serum insulin levels in the KO mice remained comparable to those in the *Fscn1* ^*+*/ *–*^ mice ( Figure 2E), with no evidence of enhanced insulin signaling, as evidenced by unaltered AKT phosphorylation at Ser473 ( Figure 2F). The insulin tolerance test (ITT) revealed mild insulin resistance in the KO mice ( Figure 2G), likely a secondary consequence of chronic hypoglycemia rather than a primary driver of metabolic dysfunction. Importantly, the hepatic expression of canonical gluconeogenic regulators ( *G6pc*, *Pck1*, *Pck2*) remained unaffected in *Fscn1*-KO mice ( Figure 2H). Following a 16-h fasting, *Fscn1*-KO mice exhibited a pronounced increase in glucose utilization (48.67% reduction vs 39.68% in *Fscn1* ^*+*/ *–*^ controls; Figure 2I), which was consistent with preserved glucose absorption but diminished reserves due to baseline hypoglycemia. Collectively, these findings exclude hyperinsulinemic and canonical gluconeogenic defects, instead implicating alternative pathways in *Fscn1* deficiency-associated hypoglycemia and growth impairment.

### *Fscn1* is involved in metabolic regulation and the glycerol-3-phosphate shuttle

**Figure 3:**
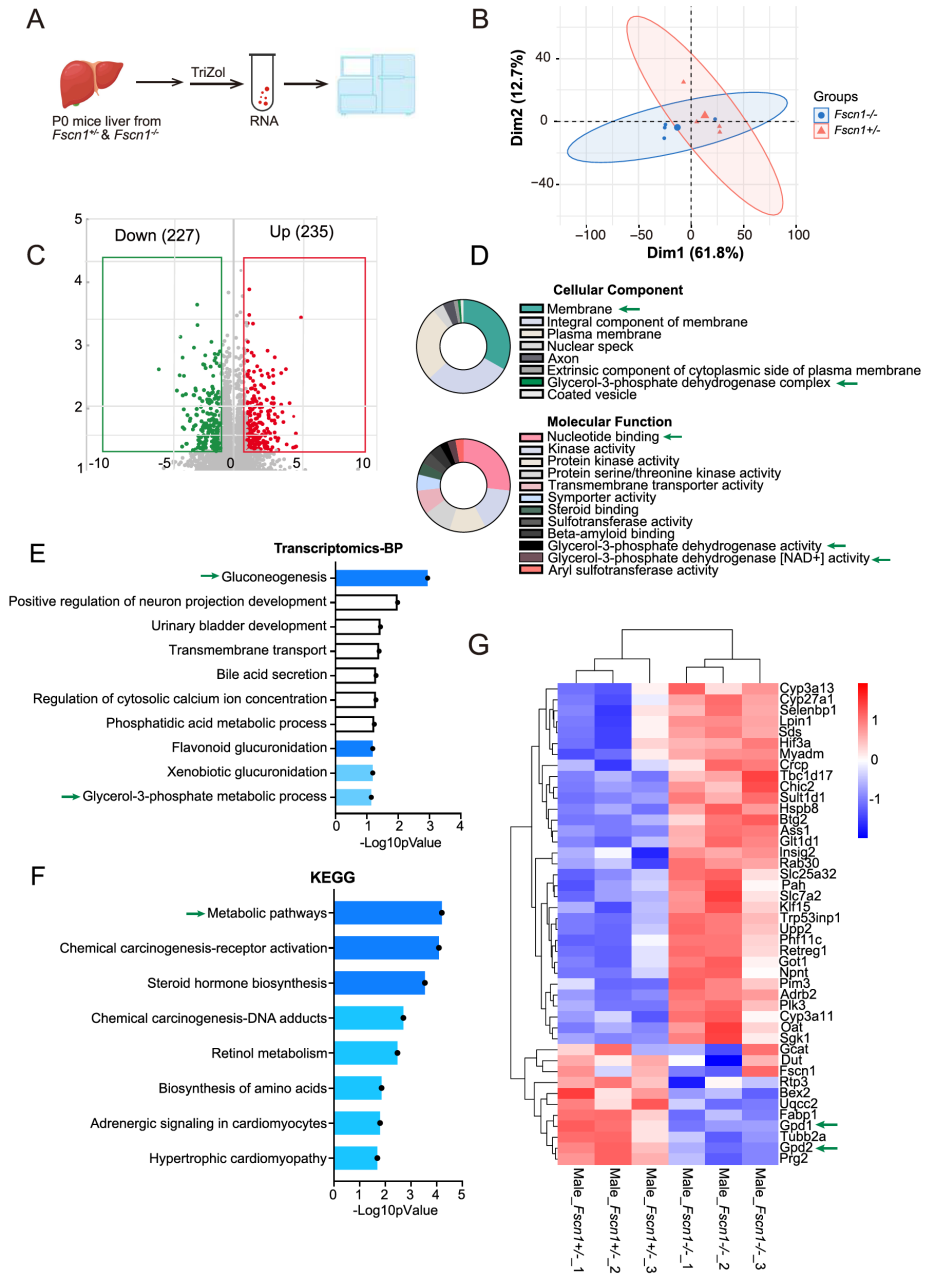
Figure 3 *Fscn1* deficiency affects the glycerol-3-phosphate process as revealed by transcriptomics analysis (A) Schematic diagram illustrating the sample preparation process for transcriptomic sequencing. (B) Principal component analysis (PCA) of the DEGs. (C) Volcano plot depicting enriched DEGs identified from transcriptomic data. (D) GO analysis of DEGs categorized into cellular components (CC) and molecular functions (MF). (E) Significance analysis of biological process (BP) terms associated with DEGs. (F) KEGG pathway enrichment analysis of DEGs in the livers of Fscn1-deficient mice. (G) Heatmap illustrating DEGs in the livers of Fscn1 +/– and Fscn1 –/– P0 mice. The mice used in the above experiments were littermates. Data are shown as the mean ± SEM, and statistical significance was determined via two-tailed t tests, with *P < 0.05, **P < 0.01, ***P < 0.001.

While *Fscn1* deficiency does not impair canonical hormone-mediated gluconeogenesis, the underlying mechanisms of hypoglycemia in KO mice remain unresolved. Immunoblotting analysis revealed a progressive postnatal decline in hepatic FSCN1 ( Supplementary Figure S2A,B), highlighting its developmental stage-specific role in neonatal metabolic adaptation. To elucidate compensatory pathways, we conducted whole-liver transcriptome profiling of *Fscn1* ^*+*/ *–*^ and *Fscn1* ^*–*/ *–*^ neonates at P0 ( Figure 3A). Principal component analysis (PCA) of the normalized RNA-seq data revealed clear genotype segregation, with principal components (PC1 and PC2) accounting for 61.8% and 12.7% of the total variance, respectively ( Figure 3B). A total of 462 DEGs were identified, with 235 genes upregulated and 227 genes downregulated in *Fscn1*-KO mice compared with *Fscn1* ^*+*/ *–*^ mice ( Figure 3C,D). Enrichment analysis of the DEGs highlighted significant Gene Ontology (GO) terms, including gluconeogenesis, nucleotide binding, and membrane dynamics ( Figure 3E,F). Kyoto Encyclopedia of Genes and Genomes (KEGG) pathway analysis revealed 18 significantly perturbed pathways ( *P*  < 0.05), including metabolic pathways, chemical carcinogenesis-receptor activation, and steroid hormone biosynthesis ( Figure 3G). Of particular interest were *Gpd1* and *Gpd2*, encoding rate-limiting enzymes of the glycerol-3-phosphate (G-3-P) shuttle—a critical system channeling cytosolic glycerol into mitochondrial gluconeogenic flux ( Figure 3D) [ 33, 43, 44] . The transcriptional downregulation of *Gpd1* and *Gpd2* in *Fscn1*-KO neonates suggests a decrease in glycerol-to-glucose conversion, a key adaptation enabling the neonatal transition from placental nutrition to autonomous metabolic regulation.

### *Fscn1* regulates glycerol-driven hepatic gluconeogenesis via the GPD1/2-mediated glycerol-3-phosphate shuttle

**Figure 4:**
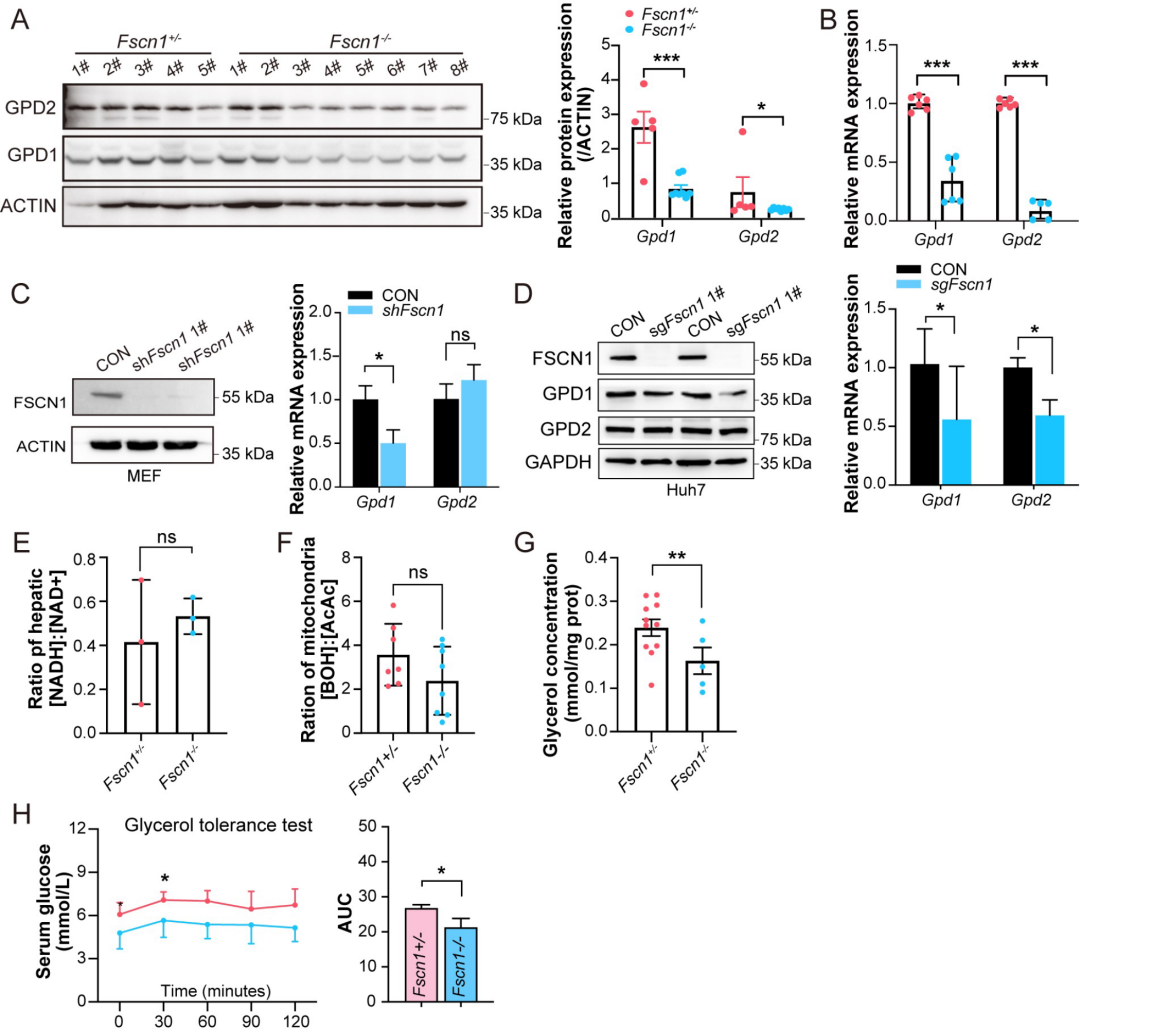
Figure 4 *Fscn1* deficiency impairs hepatic gluconeogenesis caused by glycerol (A) Western blot analysis of GPD1 and GPD2 protein levels in P0 mouse livers, with quantification using Image J. (B) mRNA levels of Gpd1 and Gpd2 in P0 mouse livers (n = 3). (C) Validation of Fscn1, GPD1, and GPD2 levels in Fscn1-knockdown MEF cells. (D) Protein and mRNA expression levels of GPD1 and GPD2 in sgFscn1-Huh7 cells. (E) NADH/NAD⁺ ratios in the livers of Fscn1 +/– and Fscn1 –/– mice at P0 were assessed via metabolomics. (F) Mitochondrial redox status measured by the BOH-to- AcAc ratio in P0 liver tissues. (G) Hepatic glycerol concentrations in Fscn1 +/– and Fscn1 –/– mice (P0). (H) Glycerol tolerance test in Fscn1 +/– and Fscn1 –/– P35 mice, with area under the curve (AUC) analysis. The mice were fasted for 16 h and administered with 2.5% glycerol (n ≥ 6). All the experiments used littermate mice. Data are presented as the mean ± SEM, statistical significance was determined via two-tailed t tests, with *P < 0.05, **P < 0.01, ***P < 0.001, and ns means P > 0.05.

To investigate the role of FSCN1 in gluconeogenesis, we focused on GPD1 and GPD2, two essential enzymes controlling the glycerol-3-phosphate (G-3-P) shuttle, which links glycerol availability to the mitochondrial redox balance [33]. *Fscn1-*KO neonates exhibited significant reductions in both hepatic GPD1 and GPD2 protein and mRNA levels ( Figure 4A,B). To further elucidate the FSCN1-dependent regulatory mechanism, we employed shRNA-mediated *Fscn1* knockdown and CRISPR-Cas9-mediated *Fscn1* knockout. shRNA targeting *Fscn1* in MEF specifically suppressed *Gpd1* transcripts without affecting *Gpd2* expression ( Figure 4C). Similarly, *Fscn1* KO in Huh7 cells recapitulated this specificity, resulting in reduced GPD1 but unchanged GPD2 ( Figure 4D). These models suggest that FSCN1 acts as a selective upstream regulator of GPD1, with GPD2 regulation appearing context dependent.

To assess the functional consequences of GPD1 deficiency, we analyzed intracellular redox dynamics and substrate utilization. Metabolomic profiling revealed a significantly elevated [NADH:NAD ^+^] ratio in *Fscn1*-KO livers ( Figure 4E), indicating an elevated cytoplasmic redox state and preserved GPD1 enzymatic activity. Additionally, *Fscn1*-KO livers accumulated VLDL-c and TG ( Supplementary Figure S2C). TG hydrolysis generates glycerol (for gluconeogenesis) and free fatty acids (for mitochondrial β-oxidation and ketogenesis). We evaluated mitochondrial function and found that *Fscn1*-KO mice presented reduced ketone body levels (BOH and AcAc; Supplementary Figure S2D), indicating impaired mitochondrial β-oxidation. Furthermore, the mitochondrial redox status was similarly disrupted in *Fscn1*-KO mice ( Figure 4F). This coordinated dysregulation of cytoplasmic and mitochondrial redox homeostasis impairs the capacity of the G-3-P shuttle to transfer reducing equivalents, as evidenced by decreased hepatic glycerol levels in *Fscn1*-KO mice ( Figure 4G).

To assess glycerol-driven gluconeogenesis directly, glycerol tolerance tests were performed on 5-week-old mice. *Fscn1*-KO mice displayed blunted glycemic responses to oral glycerol administration ( Figure 4H), confirming a defect in glycerol-to-glucose conversion. Collectively, these findings demonstrate that *Fscn1* deficiency disrupts hepatic gluconeogenesis by impairing GPD1-dependent G-3-P shuttle, ultimately leading to hypoglycemia and highlighting the essential role of FSCN1 in neonatal survival and growth.

## Discussion

Maintaining neonatal glucose homeostasis necessitates precise coordination between hepatic glucose production and peripheral utilization. In this study, we revealed an uncharacterized regulatory role for FSCN1 in regulating neonatal glucose metabolism, which is essential for neonatal survival. Unlike the canonical PCK1/PCK2-dependent gluconeogenesis pathway, FSCN1 deficiency induces hypoglycemia and growth retardation through disruption of the G-3-P shuttle, bypassing canonical insulin-regulated gluconeogenic pathways. Importantly, *Fscn1*-KO mice presented persistent hypoglycemia despite glucose supplementation rescuing neonatal lethality, accompanied by reduced body weight and lipid reserves. This metabolic defect occurs independently of hyperinsulinemia. Mechanistically, FSCN1 regulates GPD1-dependent gluconeogenesis, directly affecting the glycerol-to-glucose conversion process. Our findings identify FSCN1 as a novel metabolic regulator and highlight a critical link between cytoskeletal dynamics and hepatic glucose production, potentially providing insights into congenital hypoglycemia disorders associated with cytoskeletal dysfunction.

Neonatal hypoglycemia affects approximately 10% of healthy term infants, predominantly within the first 24–48 h post-birth [ 45, 46] , and is attributable to high brain glucose demands and a large brain-to-body mass ratio [47]. While hypoglycemia can often be asymptomatic or present with nonspecific symptoms, severe cases (defined as blood glucose levels less than 40 mg/dL in term infants) can result in life-threatening complications, including cardiac arrhythmias, seizures, and irreversible neurological damage [ 48, 49] . Prompt glucose supplementation is critical to prevent a cerebral energy crisis because the brain preferentially relies on glucose over ketones during acute hypoglycemia [50]. In our model, *Fscn1*-KO neonates exhibited 52.2% lethality within 24 h, indicating impaired postnatal metabolic adaptation. Clinical thresholds for hypoglycemia vary across etiologies [51]; however, *Fscn1*-KO neonates presented significantly lower blood glucose levels (3.76±1.47 mM) than their heterozygous counterparts did (5.57±0.77 mM at P0), which is below the mammalian physiological threshold (~5.0 mM) [52] and aligns with the diagnostic criteria of pathogenic hypoglycemia in human neonates [51]. The survival of *Fscn1*-KO pups following oral glucose rescue underscores their glucose dependency, phenocopying starvation-induced hypoglycemia. This dependency is further evidenced by reduced hepatic ATP levels (1.05±0.57 mM/L/mg prot) and compensatory AMPK activation in *Fscn1*-KO pups, indicative of systemic energy deprivation. FFA, which undergoes β-oxidation to generate ketone bodies for cerebral energy supply during fasting [29], also enhances gluconeogenic flux by providing acetyl-CoA and NADH for glucose synthesis [ 53, 54] . The concurrent reduction in ketone body levels in *Fscn1*-deficient neonates, a direct metabolite of impaired FFA utilization, further corroborates systemic energy deficiency. These findings collectively emphasize the non-redundant role of glucose in neonatal energy homeostasis, particularly under conditions of cytoskeletal dysregulation.

Our investigation into hepatic gluconeogenesis revealed that *Fscn1* KO did not impede the canonical hormone-regulated transcriptional gluconeogenic pathway, thereby excluding hyperinsulinemic hypoglycemia as a potential etiology. Circulating-insulin concentrations and downstream insulin signaling targets AKT phosphorylation status remained unaltered in *Fscn1*-KO neonates. Transcriptomic analysis revealed the selective disruption of glycerol-mediated gluconeogenesis, notably Gpd1 and Gpd2, which is an alternative metabolic route governed by substrate availability and intracellular redox homeostasis, in KO mice [24]. Systematic validation across *in vitro* and *in vivo* models revealed that *Fscn1* deficiency significantly downregulates GPD1 expression, perturbs the mitochondrial and cytoplasmic [NADH:NAD ^+^] redox equilibrium, and reduces hepatic glycerol availability. The functional deficit in glycerol-to-glucose conversion was further confirmed by impaired glycemic recovery in *Fscn1*-KO mice during glycerol tolerance tests. Intriguingly, while both *Fscn1*-KO mice and derived cell lines presented reduced Gpd1 mRNA and protein expression levels, the regulatory interplay between FSCN1 and GPD1 appears to be indirect because of their distinct subcellular localization.

FSCN1 is considered a marker of immature hepatocytes [55], and its expression level progressively decreases during early liver development [56]. Notably, impaired hepatic glyceroneogenesis in *Fscn1*-KO mice was not associated with hepatocyte injury. However, perivascular nuclear aggregation of hepatocytes adjacent to portal veins, a histological feature suggestive of subclinical injury [57], was detected in *Fscn1*-KO neonates. Quantitative assessment of liver function enzyme levels, including alanine aminotransferase (ALT) and aspartate transaminase (AST) levels and the AST/ALT ratio ( Supplementary Figure S2E,F), revealed no evidence of substantial hepatic damage. These findings collectively reinforce the notion that *Fscn1* KO induces hypoglycemia through selective disruption of the glycerol-gluconeogenesis pathway rather than through impairment of hepatocyte structure. This study highlights the critical role of FSCN1 in early hepatic gluconeogenesis, suggesting that metabolic regulation during liver development has been underestimated, which might provide a clinical reference for metabolic abnormalities caused by cytoskeletal deletions.

In conclusion, we redefined FSCN1 as a linchpin connecting actin dynamics to neonatal energy homeostasis. By orchestrating the glycerol gluconeogenic shunt, FSCN1 ensures glucose output during the critical postnatal transition, offering novel insights into congenital hypoglycemia and cytoskeletal‒metabolic crosstalk. These findings pave the way for exploring cytoskeletal targets in metabolic disorders and refining neonatal hypoglycemia management strategies.

## Supplementary Data

Supplementary data is available at *Acta Biochimica et Biophysica Sinica* online.

## References

[1] Lamb MC, Tootle TL (2020). Fascin in cell migration: more than an actin bundling protein. Biology.

[2] Lin S, Taylor MD, Singh PK, Yang S (2021). How does fascin promote cancer metastasis?. FEBS J.

[3] Sarantelli E, Mourkakis A, Zacharia LC, Stylianou A, Gkretsi V (2023). Fascin-1 in cancer cell metastasis: old target-new insights. Int J Mol Sci.

[4] Parker AL, Kavallaris M, McCarroll JA (2014). Microtubules and their role in cellular stress in cancer. Front Oncol.

[5] Elkhatib N, Neu MB, Zensen C, Schmoller KM, Louvard D, Bausch AR, Betz T (2014). Fascin plays a role in stress fiber organization and focal adhesion disassembly. Curr Biol.

[6] Harker AJ, Katkar HH, Bidone TC, Aydin F, Voth GA, Applewhite DA, Kovar DR (2019). Ena/VASP processive elongation is modulated by avidity on actin filaments bundled by the filopodia cross-linker fascin. Mol Biol Cell.

[7] Winkelman JD, Bilancia CG, Peifer M, Kovar DR (2014). Ena/VASP Enabled is a highly processive actin polymerase tailored to self-assemble parallel-bundled F-actin networks with Fascin. Proc Natl Acad Sci USA.

[8] Villari G, Jayo A, Zanet J, Fitch B, Serrels B, Frame M, Stramer BM (2015). A direct interaction between fascin and microtubules contributes to adhesion dynamics and cell migration. J Cell Sci.

[9] Jayo A, Malboubi M, Antoku S, Chang W, Ortiz-Zapater E, Groen C, Pfisterer K (2016). Fascin regulates nuclear movement and deformation in migrating cells. Dev Cell.

[10] Hashimoto Y, Kim DJ, Adams JC (2011). The roles of fascins in health and disease. J Pathol.

[11] Lin S, Li Y, Wang D, Huang C, Marino D, Bollt O, Wu C (2021). Fascin promotes lung cancer growth and metastasis by enhancing glycolysis and PFKFB3 expression. Cancer Lett.

[12] Lawson CD, Peel S, Jayo A, Corrigan A, Iyer P, Baxter Dalrymple M, Marsh RJ (2022). Nuclear fascin regulates cancer cell survival. eLife.

[13] Yoder BJ, Tso E, Skacel M, Pettay J, Tarr S, Budd T, Tubbs RR (2005). The expression of fascin, an actin-bundling motility protein, correlates with hormone receptor–negative breast cancer and a more aggressive clinical course. Clin Cancer Res.

[14] Han X, Du S, Chen X, Min X, Dong Z, Wang Y, Zhu C (2023). Lactate-mediated Fascin protrusions promote cell adhesion and migration in cervical cancer. Theranostics.

[15] Darnel AD, Behmoaram E, Vollmer RT, Corcos J, Bijian K, Sircar K, Su J (2009). Fascin regulates prostate cancer cell invasion and is associated with metastasis and biochemical failure in prostate cancer. Clin Cancer Res.

[16] Fu H, Hu Z, Wen J, Wang K, Liu Y (2009). TGF-β promotes invasion and metastasis of gastric cancer cells by increasing fascin1 expression via ERK and JNK signal pathways. Acta Biochim Biophys Sin.

[17] Kang J, Wang J, Yao Z, Hu Y, Ma S, Fan Q, Gao F (2018). Fascin induces melanoma tumorigenesis and stemness through regulating the Hippo pathway. Cell Commun Signal.

[18] AlMalki RH, Al-Nasrallah HK, Aldossry A, Barnawi R, Al-Khaldi S, Almozyan S, Al-Ansari MM (2024). Comparative analysis of breast cancer metabolomes highlights fascin’s central role in regulating key pathways related to disease progression. Int J Mol Sci.

[19] Wu M, Hao Y, Wu X, Zhu M, Chen X, Qi J, Yu Z (2024). SirT7-mediated transcription of fascin in hyperglycemic glomerular endothelial cells contributes to EndMT in diabetic nephropathy. Acta Biochim Biophys Sin.

[20] De Arcangelis A, Georges-Labouesse E, Adams JC (2004). Expression of fascin-1, the gene encoding the actin-bundling protein fascin-1, during mouse embryogenesis. Gene Expression Patterns.

[21] Zhang FR, Tao LH, Shen ZY, Lv Z, Xu LY, Li EM (2008). Fascin expression in human embryonic, fetal, and normal adult tissue. J Histochem Cytochem.

[22] Yamakita Y, Matsumura F, Yamashiro S (2009). Fascin1 is dispensable for mouse development but is favorable for neonatal survival. Cell Motil Cytoskeleton.

[23] Al-Khaldi S, Almohanna F, Barnawi R, Fallatah M, Islam SS, Ghebeh H, Al-Alwan M (2022). Fascin is essential for mammary gland lactogenesis. Dev Biol.

[24] Petersen MC, Vatner DF, Shulman GI (2017). Regulation of hepatic glucose metabolism in health and disease. Nat Rev Endocrinol.

[25] Zhang X, Yang S, Chen J, Su Z (2018). Unraveling the regulation of hepatic gluconeogenesis. Front Endocrinol.

[26] Han HS, Kang G, Kim JS, Choi BH, Koo SH (2016). Regulation of glucose metabolism from a liver-centric perspective. Exp Mol Med.

[27] Zhao Y, Li S, Chen Y, Wang Y, Wei Y, Zhou T, Zhang Y (2023). Histone phosphorylation integrates the hepatic glucagon-PKA-CREB gluconeogenesis program in response to fasting. Mol Cell.

[28] Aprille JR, Yaswen P, Rulfs J (1981). Acute postnatal regulation of pyruvate carboxylase activity by compartmentation of mitochondrial adenine nucleotides. Biochim Biophys Acta.

[29] Girard J, Ferre P, Pegorier JP, Duee PH (1992). Adaptations of glucose and fatty acid metabolism during perinatal period and suckling-weaning transition. Physiol Rev.

[30] Kalhan SC, Mahajan S, Burkett E, Reshef L, Hanson RW (2001). Glyceroneogenesis and the source of glycerol for hepatic triacylglycerol synthesis in humans. J Biol Chem.

[31] Gao Y, Dong R, Yan J, Chen H, Sang L, Yao X, Fan D (2024). Mitochondrial deoxyguanosine kinase is required for female fertility in mice. Acta Biochim Biophys Sin.

[32] Miyawaki S, Kuroki S, Maeda R, Okashita N, Koopman P, Tachibana M (2020). The mouse
*Sry* locus harbors a cryptic exon that is essential for male sex determination. Science.

[33] Madiraju AK, Erion DM, Rahimi Y, Zhang XM, Braddock DT, Albright RA, Prigaro BJ (2014). Metformin suppresses gluconeogenesis by inhibiting mitochondrial glycerophosphate dehydrogenase. Nature.

[34] Hao Q, Dong R, Bai W, Chang D, Yao X, Zhang Y, Xu H (2024). Screening for metastasis-related genes in mouse melanoma cells through sequential tail vein injection. Biophys Rep.

[35] He YJ, Qin Y, Zhang TL, Zhu YY, Wang ZJ, Zhou ZS, Xie TZ (2021). Migration of (non-) intentionally added substances and microplastics from microwavable plastic food containers. J Hazard Mater.

[36] Sun J, Feng Q, He Y, Wang M, Wu Y (2024). Lactate activates CCL18 expression via H3K18 lactylation in macrophages to promote tumorigenesis of ovarian cancer. Acta Biochim Biophys Sin.

[37] Liu X, Mi X, Wang Z, Zhang M, Hou J, Jiang S, Wang Y (2020). Ginsenoside Rg3 promotes regression from hepatic fibrosis through reducing inflammation-mediated autophagy signaling pathway. Cell Death Dis.

[38] Bai W, Hao Q, Zhang Z, Han B, Xiao H, Chang D, Zhu Y,
*et al*. Identification of a novel inflammation-related gene signature for predicting inflammatory breast cancer survival.
Genome Instability & Disease 2023, 4(3): 154–175. https://link.springer.com/article/10.1007/s42764-023-00102-8.

[39] Lin SC, Hardie DG (2018). AMPK: sensing glucose as well as cellular energy status. Cell Metab.

[40] Luo J, Yang H, Song BL (2020). Mechanisms and regulation of cholesterol homeostasis. Nat Rev Mol Cell Biol.

[41] Nowaczyk MJM, Irons MB (2012). Smith–Lemli–Opitz syndrome: phenotype, natural history, and epidemiology. Am J Med Genet C Semin Med Genet.

[42] Rozenkova K, Güemes M, Shah P, Hussain K (2015). The diagnosis and management of hyperinsulinaemic hypoglycaemia. Jcrpe.

[43] Harding Jr JW, Pyeritz EA, Copeland ES, White 3rd HB (1975). Role of glycerol 3-phosphate dehydrogenase in glyceride metabolism. Effect of diet on enzyme activities in chicken liver. Biochem J.

[44] Harding Jr JW, Pyeritz EA, Morris HP, White 3rd HB (1975). Proportional activities of glycerol kinase and glycerol 3-phosphate dehydrogenase in rat hepatomas. Biochem J.

[45] Arya VB, Senniappan S, Guemes M, Hussain K (2014). Neonatal hypoglycemia. Ind J Pediatr.

[46] Saudubray JM, Marsac C, Limal JM, Dumurgier E, Charpentier C, Ogier H, Coudè FX (1981). Variation in plasma ketone bodies during a 24-hour fast in normal and in hypoglycemic children: Relationship to age. J Pediatr.

[47] Dani C, Corsini I (2020). Guidelines for management of neonatal hypoglycemia. JAMA Pediatr.

[48] Gerich JE (2000). Physiology of glucose homeostasis. Diabetes Obesity Metab.

[49] Aynsley-Green A (2000). Practical management of hyperinsulinism in infancy. Arch Dis Child Fetal Neonatal Ed.

[50] Gerich JE (2010). Role of the kidney in normal glucose homeostasis and in the hyperglycaemia of diabetes mellitus: therapeutic implications. Diabetic Med.

[51] Blanco CL, Kim J (2022). Neonatal glucose homeostasis. Clin Perinatology.

[52] Liu J, Wang X, Zhang W, Liao G, Shao Z, Brosius J, Deng C (2023). Evolution of GCGR family ligand-receptor extensive cross-interaction systems suggests a therapeutic direction for hyperglycemia in mammals. Acta Biochim Biophys Sin.

[53] Pegorier JP, Ferre P, Girard J (1977). The effects of inhibition of fatty acid oxidation in suckling newborn rats. Biochem J.

[54] Ferre P, Pegorier JP, Williamson DH, Girard J (1979). Interactions
*in vivo* between oxidation of non-esterified fatty acids and gluconeogenesis in the newborn rat. Biochem J.

[55] Gest C, Sena S, Dif L, Neaud V, Loesch R, Dugot-Senant N, Paysan L (2023). Antagonism between wild-type and mutant β-catenin controls hepatoblastoma differentiation via fascin-1. JHEP Rep.

[56] Hayashi Y, Toda K, Saibara T, Okamoto S, Osanai M, Enzan H, Lee GH (2008). Expression of fascin-1, an actin-bundling protein, in migrating hepatoblasts during rat liver development. Cell Tissue Res.

[57] Li W, Yang L, He Q, Hu C, Zhu L, Ma X, Ma X (2019). A Homeostatic arid1a-dependent permissive chromatin state licenses hepatocyte responsiveness to liver-injury-associated YAP signaling. Cell Stem Cell.

